# High rates of placental inflammation among samples collected by the Multi-Omics for Mothers and Infants consortium

**DOI:** 10.1016/j.ajog.2024.04.034

**Published:** 2025-02

**Authors:** Joshua F. Robinson, Sayan Das, Waqasuddin Khan, Rasheda Khanam, Joan T. Price, Anisur Rahman, Salahuddin Ahmed, Said Mohammed Ali, Saikat Deb, Brian Deveale, Arup Dutta, Matthew Gormley, Steven C. Hall, A.S.M. Tarik Hasan, Aneeta Hotwani, Mohamed Hamid Juma, Margaret P. Kasaro, Javairia Khalid, Pallavi Kshetrapal, Michael T. McMaster, Usma Mehmood, Imran Nisar, Jesmin Pervin, Sayedur Rahman, Rubhana Raqib, Ali San, Protim Sarker, Sami T. Tuomivaara, Ge Zhang, Yan Zhou, Shaki Aktar, Abdullah H. Baqui, Fyezah Jehan, Sunil Sazawal, Jeffrey S.A. Stringer, Susan J. Fisher

**Affiliations:** a Department of Obstetrics, Gynecology, and Reproductive Sciences, Center for Reproductive Sciences, University of California, San Francisco, CA; b Public Health Laboratory Ivo de Carneri, Wawi, Chake, Pemba, Zanzibar, Tanzania; c Biorepository and Omics Research Group, Department of Pediatrics and Child Health, Faculty of Health Sciences, Medical College, The Aga Khan University, Stadium Road, Karachi, Pakistan; d Department of Pediatrics and Child Health, Faculty of Health Sciences, Medical College, The Aga Khan University, Stadium Road, Karachi, Pakistan; e Department of International Health, Johns Hopkins Bloomberg School of Public Health, Baltimore, MD; f UNC Global Projects – Zambia, Lusaka, Zambia; g Department of Obstetrics and Gynecology, University of North Carolina School of Medicine, Chapel Hill, NC; h Maternal and Child Health Division, International Centre for Diarrhoeal Disease Research Bangladesh, Dhaka, Bangladesh; i Projahnmo Research Foundation, Dhaka, Bangladesh; j Center for Public Health Kinetics, Vinoba Puri, Lajpatnagar II, New Delhi, India; k Eli and Edythe Broad Center for Regeneration Medicine and Stem Cell Research, University of California, San Francisco, CA; l Department of Urology, University of California, San Francisco, San Francisco, CA; m Sandler-Moore Mass Spectrometry Core Facility, University of California, San Francisco, San Francisco, CA; n Department of Gynaecology and Obstetrics, University of Zambia School of Medicine, Lusaka, Zambia; o Maternal and Child Health, Translational Health Science and Technology Institute, Faridabad, India; p Department of Cell and Tissue Biology, University of California, San Francisco, San Francisco, CA; q International Centre for Diarrhoeal Disease Research Bangladesh, Dhaka, Bangladesh; r Nutrition and Clinical Services Division, International Centre for Diarrhoeal Disease Research Bangladesh, Dhaka, Bangladesh; s Division of Human Genetics, Cincinnati Children’s Hospital Medical Center, Department of Pediatrics, University of Cincinnati College of Medicine, Cincinnati, OH; t Center for Prevention of Preterm Birth, Perinatal Institute, Cincinnati Children’s Hospital Medical Center, Cincinnati, OH; u March of Dimes Prematurity Research Center Ohio Collaborative, Cincinnati Children’s Hospital Medical Center and Department of Pediatrics, University of Cincinnati College of Medicine, Cincinnati, OH

**Keywords:** chorionic villi, chronic villitis, inflammation, parturition, placenta, pregnancy, prematurity preterm birth, transcriptomics

## Abstract

**Background:**

The Multi-Omics for Mothers and Infants consortium aims to improve birth outcomes. Preterm birth is a major obstetrical complication globally and causes significant infant and childhood morbidity and mortality.

**Objective:**

We analyzed placental samples (basal plate, placenta or chorionic villi, and the chorionic plate) collected by the 5 Multi-Omics for Mothers and Infants sites, namely The Alliance for Maternal and Newborn Health Improvement Bangladesh, The Alliance for Maternal and Newborn Health Improvement Pakistan, The Alliance for Maternal and Newborn Health Improvement Tanzania, The Global Alliance to Prevent Prematurity and Stillbirth Bangladesh, and The Global Alliance to Prevent Prematurity and Stillbirth Zambia. The goal was to analyze the morphology and gene expression of samples collected from preterm and uncomplicated term births.

**Study Design:**

The teams provided biopsies from 166 singleton preterm (<37 weeks’ gestation) and 175 term (≥37 weeks’ gestation) deliveries. The samples were fixed in formalin and paraffin embedded. Tissue sections from these samples were stained with hematoxylin and eosin and subjected to morphologic analyses. Other placental biopsies (n=35 preterm, 21 term) were flash frozen, which enabled RNA purification for bulk transcriptomics.

**Results:**

The morphologic analyses revealed a surprisingly high rate of inflammation that involved the basal plate, placenta or chorionic villi, and the chorionic plate. The rate of inflammation in chorionic villus samples, likely attributable to chronic villitis, ranged from 25% (Pakistan site) to 60% (Zambia site) of cases. Leukocyte infiltration in this location vs in the basal plate or chorionic plate correlated with preterm birth. Our transcriptomic analyses identified 267 genes that were differentially expressed between placentas from preterm vs those from term births (123 upregulated, 144 downregulated). Mapping the differentially expressed genes onto single-cell RNA sequencing data from human placentas suggested that all the component cell types, either singly or in subsets, contributed to the observed dysregulation. Consistent with the histopathologic findings, gene ontology analyses highlighted the presence of leukocyte infiltration or activation and inflammatory responses in both the fetal and maternal compartments.

**Conclusion:**

The relationship between placental inflammation and preterm birth is appreciated in developed countries. In this study, we showed that this link also exists in developing geographies. In addition, among the participating sites, we found geographic- and population-based differences in placental inflammation and preterm birth, suggesting the importance of local factors.

## Introduction

Preterm birth (PTB), defined as live delivery before 37 completed weeks of gestation, is a leading cause of morbidity and mortality in children under the age of 5 years. An estimated 15 million babies are born prematurely every year, equating to a rate of about 11% globally.^1^ The frequency varies from 5% to18% across 184 countries and is increasing in nearly all of them.^1^ About 1 million children die each year because of PTB, accounting for 18% of all deaths among children (aged <5 years) and for 35% of all deaths among newborns (aged <28 days).^2^ The fatality rate is much higher in low-income countries because of the lower availability of postnatal care such as warmth, breastfeeding support, and basic interventions for infections and breathing difficulties.^1^ Surviving babies are at increased risk for sudden infant death syndrome, early-life infections, and adverse respiratory outcomes.^3^^,^^4^ Long-term consequences of PTB include inflammatory, cardiovascular, pulmonary, renal, metabolic, and neurodevelopmental disorders.^5^^,^^6^ Consequently, a large number of lives and disability-adjusted life years (DALY) are lost because of PTB. In 2019, PTB was responsible (worldwide) for 126,752 deaths and 11.3 million DALYs, two-thirds of which were in Western Sub-Saharan Africa and South Asia.^7^

**AJOG at a Glance**

**Why was this study conducted?**

Preterm birth is a major obstetrical complication globally that causes significant infant and childhood morbidity and mortality. Its causes remain poorly understood.

**Key findings**

Analyses of placental biopsies collected in Bangladesh, Zambia, Tanzania, and Pakistan revealed a surprisingly high rate of inflammation. Leukocyte infiltration of chorionic villi, chronic villitis, correlated with preterm birth. The incidence in this study, which ranged from 25% (Pakistan site) to 62% (Zambia site) of cases, was higher than what has been reported in the United States for preterm births (17%).

**What does this add to what is known?**

These data suggest geographic- and population-based differences in placental inflammation and the possible triggers of preterm birth.

The prevention of PTB is a significant priority for achieving the United Nations Sustainable Development Goal 3 target #3.2, which aims to end all preventable deaths of newborns and children <5 years of age by 2030. One of the greatest challenges to achieving this goal is the lack of a unified understanding of the pathogenesis of PTB, which has a complex etiology that consists of multiple pathways. Recognized risk factors include multiparity; antepartum hemorrhage; multiple miscarriages or abortions; previous PTB(s); preexisting maternal diseases; preeclampsia; genetic factors; drug or alcohol abuse; problems with the uterus, cervix, or placenta; and ascending or systemic infections during pregnancy.^8^, ^9^, ^10^, ^11^, ^12^ The fact that, in many cases, the specific causes and risk factors are unknown is a further complication. In addition, it is difficult to gain insights by contextualizing this condition because the mechanisms that lead to normal birth are poorly understood.

Among multifactorial etiologies, ascending infection (from the vagina to the uterus) is most strongly associated with PTB. Although the specific pathophysiology of infection-related preterm labor (PTL) remains poorly understood, the ability of pathogens to cross the cervical and placental or fetal membrane barrier is well established.^13^ Many studies have shown that the host vaginal or cervical microbiota trigger inflammatory cascades that may increase the risk for cervical ripening, premature rupture of membranes, and PTB.^13^, ^14^, ^15^, ^16^ Numerous studies have reported that PTB cases have more lesions than term cases, including umbilical cord vasculitis, decidual vascular abnormalities, and chronic villitis.^17^

In this study, the Multi-Omics for Mothers and Infants (MOMI) consortium analyzed placental samples from preterm and normal term births collected at the 5 MOMI sites, namely The Alliance for Maternal and Newborn Health Improvement (AMANHI) Bangladesh, AMANHI Pakistan, AMANHI Tanzania, the Global Alliance to Prevent Prematurity and Stillbirth (GAPPS) Bangladesh, and GAPPS Zambia. These cohorts were assembled to establish biorepositories of samples and phenotypic data to study maternal and fetal outcomes from diverse populations of South Asia and sub-Saharan Africa.^18^^,^^19^ A major objective was to determine if the link between placental inflammation and PTB, well established in developed countries, was also evident in these developing geographies.

## Materials and Methods

### Placental tissue collection

Placental biopsies were obtained according to the standard operating procedures (SOPs) developed by the AMANHI^18^ or GAPPS (https://www.gapps.org/Home/SOPsAndRelatedMaterials) collaboratives. All pregnant women provided written informed consent for participation in the original study and for future use of specimens. All tissues were collected according to protocols approved for each collection site as follows: for Pakistan, The Aga Khan University Ethical Review Committee and the Pakistan National Bioethics Committee approved the protocols; for Zambia, the University of Zambia, The Zambian Ministry of Health National Health Research Authority, and the University of North Carolina at Chapel Hill approved the protocols; for Tanzania, the Zanzibar Medical Research Ethics Committee (ZAMAREC) approved the protocols; and for Bangladesh, the Ethical Review Committee of the International Centre for Diarrhoeal Disease Research Bangladesh and the Johns Hopkins Bloomberg School of Public Health Institutional Review Board approved the protocols. For the reported studies, ethical exemptions were sought from the respective country institutional review boards and regulated under the necessary material transfer and data transfer agreements.

Briefly, placentas were biopsied (10 mm punch) within 1 hour of delivery. If a suitable sample could not be obtained from a preterm specimen, a 0.5 cm biopsy was taken with scissors or a scalpel. Using one of these methods, full thickness specimens that included the maternal and fetal aspects were obtained. The location of the biopsies was specified in the individual study SOPs with 2 sites being separated by several centimeters on the placental disc, avoiding the site of umbilical cord insertion and the disc edge. After washing in sterile phosphate buffered saline (PBS), specimens for histopathology were submerged in a 50 mL container of 10% neutral buffered formalin (Sigma-Aldrich, St. Louis, MO). After 48 to 72 hours, the biopsies were transferred to 70% histology grade ethanol (Sigma-Aldrich). The samples were kept at 4°C until they were embedded in paraffin. The blocks were stored at room temperature before shipping to the University of California San Francisco (UCSF). An additional full-thickness biopsy was taken in an area adjacent to the histopathology specimen, washed in sterile PBS, and flash frozen. The samples were maintained at 4˚C before they were stored at −80°C before shipping on dry ice to UCSF.

### Morphologic analyses

We received biopsies from 294 preterm (gestation <37 weeks) and 291 term (gestation ≥37 weeks) births. Because of technical difficulties, the ultimate number analyzed was 166 spontaneous, singleton preterm (<37 weeks) and 175 term (≥37 weeks) deliveries. The subject data for the participants are summarized in Supplemental Table 1. Among the specimens, there were 20 induced births; Zambia had 16 (3 term, 13 PTB with 5 at <34 weeks and 8 at >34 weeks); Pakistan had 2 (PTB, >34 weeks); and Tanzania had 2 (1 term, 1 PTB >34 weeks). Formalin-fixed, paraffin-embedded biopsies were sectioned and stained with hematoxylin and eosin (H & E). The slides were examined and photographed using a standard light microscope (Leica DM 5000B with a DFC 450 color camera). Ten randomly chosen fields were analyzed and scored by 2 independent observers who were blinded to the case-control status.

Six endpoints, some with multiple features, were assessed for each sample as follows^20^:

(1) The presence of basal plate (BP), chorionic villi (CV), and chorionic plate (CP)
(2) Villus, syncytiotrophoblasts (STB), and cytotrophoblast (CTB) changes
  – hypoplastic terminal villi
  – Tenney Parker changes
  – STB lesions (sloughing or aggregation)
  – CTB aggregation in the BP
  – abnormal CTB morphology in the BP
  – CTB aggregation in the CP
(3) Inflammation
  – increased number of Hofbauer cells in villus core
  – increased number of monocytes in blood vessels of stem villi or CP
  – leukocytes in the CP
  – leukocytes in blood vessel walls of the CP
  – leukocytes in the BP
(4) Blood vessel changes
  – decreased blood vessels in terminal villi
  – increased blood vessels in terminal villi
  – bleeding in floating villi
  – bleeding in the BP
  – bleeding in the CP
  – coagulation
(5) Villous core edema
(6) Avascular fibrotic villi

The endpoints listed above were assessed across all the samples. A scoring system was developed in which a pathologic feature was either present (designated by a 1) or absent (designated by a 0). Scores were summed and averaged for the 3 endpoints with multiple features, namely (1) villus, STB, or CTB changes; (2) inflammation; and (3) blood vessel changes. Representative and unusual findings were photographed.

### Transcriptomics

RNA was isolated from the flash frozen biopsies by using a spin column–based nucleic acid purification approach. Briefly, frozen specimens were dissected free of CPs and BPs, which were inconsistently present in the samples. They were placed in 2 mL tubes with 2.8 mm ceramic beads (Fisherbrand) and 600 μL lysis buffer (RLTplus; Qiagen, Waltham, MA) containing 1% (v/v) β-mercaptoethanol (Sigma-Aldrich) and 0.5% (v/v) Reagent DX (Qiagen, Redwood City, CA). Samples were then homogenized at 4°C using a Bead Mill 24 device (Fisherbrand), which enabled delivery of two 20 second pulses with a 10 second rest in between. Debris was pelleted by centrifugation at 15,000×g for 2 minutes (Eppendorf 5415 R microcentrifuge). The supernatants were transferred to 1.5 mL tubes (Eppendorf, Enfield, CT) and the centrifugation step was repeated. Total RNA was purified following the manufacturer’s protocol (Qiagen RNeasy Plus Mini Kit). Absorbance at 280, 260, and 230 nm was measured (Nanodrop 2000; Thermo Scientific, Waltham, MA) to determine the concentration and purity; 5 μL was aliquoted to measure integrity, and the remaining sample was stored at −80°C.

RNA integrity was determined using microfluidic electrophoresis (Agilent Tapestation 4200, Santa Clara, CA) with either an RNA ScreenTape assay or high-sensitivity ScreenTape assay depending on the concentration. Aliquots with concentrations exceeding the dynamic range were diluted to 500 ng/μL immediately before the assay, which was performed using the manufacturer’s protocol. An RNA integrity number (RIN) was automatically assigned on a scale of 0 to 10 according to a standardized assessment of the electrophoretic trace of the 18S and 28S ribosomal RNAs (rRNAs). Samples with a RIN ≥7 were sequenced (RNA-seq). Briefly, a portion of each sample (500 ng) was used for complimentary DNA (cDNA) synthesis and library construction. A genomic DNA cleanup step was performed to remove contaminants. Next, libraries were prepared using the Kapa Biosystems adapter kit. RNA-seq was performed on a 150PE NovaSeq S4 flowcell to obtain ∼25 million reads per sample.

We implemented the established Nextflow nf-core rnaseq pipeline (v. 3.2) to obtain transcript counts from raw FASTQ files. In brief, quality control was performed on merged FASTQ files using FastQC (v. 0.11.9). To process the raw RNA-seq reads into transcript counts, we performed adapter and quality-based trimming (Trim Galore! v. 0.6.6), removed genomic (BBSplit) and rRNA contaminants (SortMeRNA v. 4.2.0), and aligned reads (STAR, v. 2.6.1b; GRCh38/hg38). SAMtools (v. 1.10) and Picard (v. 2.18.23) were used for intermediate processing steps, including sorting and indexing of aligned reads and marking, respectively. Expression of each transcript was then quantified (Salmon v. 1.4.0). Additional quality control was performed using RSeQC (v. 3.0.0), Qualimap (v. 2.2.2d), dupRadar (v. 1.18.0), Preseq, and DESeq2 (v. 1.28.0). Six samples were removed from the analysis because of insufficient reads, which reduced the total number of samples analyzed to 56. The subject data for the participants are summarized in Supplemental Table 2. Among the cases there were 3 induced births, namely 1 PTB (>34 weeks) in Zambia and 2 PTBs (>34 weeks) in Tanzania. We performed dimensionality reduction using Uniform Manifold Approximation and Projection (UMAP) analysis to evaluate the impact of the collection site and gestational age.^21^ The UMAP analysis was conducted in R using default R-implementation (R Core Team, Vienna, Austria) (https://cran.rproject.org/web/packages/umap/vignettes/umap.html).

Volcano plots were generated using VolcaNoseR.^22^ Raw (FASTQ) and normalized counts were deposited in the National Center for Biotechnology Information (NCBI) Gene Expression Omnibus (GSE240306) and the Gates Foundation Synapse website.

To identify differentially expressed (DE) genes between placental samples from term (≥37.0 weeks’ gestation) and those from preterm delivery (<37.0 weeks’ gestation) cases, we applied DESeq2 (v. 1.28.0) to normalize the data, which accounted for variations in sequencing depth and RNA composition. We focused on the subset of 11,444 genes that were expressed in the majority of samples. First, we implemented a cutoff of *P*<.05 (Bonferroni-corrected) to identify significantly DE genes. Because no genes were identified, we incorporated less stringent significance criteria (uncorrected *P*<.05) and an absolute fold-difference of ≥1.25 into the analysis.

We performed functional analysis of DE genes to identify enriched gene ontology (GO) terms (Biological Processes, Level 4) using the Database for Annotation, Visualization and Integrated Discovery (DAVID v. 6.9),^23^ a significance criteria of *P*<.001, and a minimum number of DE genes (>8 per GO term).

### Quantitative reverse transcriptase–polymerase chain reaction validation of differentially expressed genes

We used quantitative reverse transcriptase–polymerase chain reaction (qRT-PCR) to confirm differential expression of DE genes in cases (n=36) and controls (n=20). Most (51/56) were aliquots remaining after the RNA-seq analysis but were supplemented with additional MOMI samples. The targets were as follows: ankyrin repeat and SOCS box protein 2 (*ASB2*), neurofascin (*NFASC*), C-X-C motif chemokine ligand 1 (*CXCL1*), angiopoietin 2 (*ANGPT2*), SERTA domain containing 4 (*SERTAD4*), and carbonic anhydrase 1 (*CA1*). Aliquots of purified RNA (300 ng) were converted to cDNA (Quantabio qScript cDNA Synthesis Kit, Beverly, MA). We conducted qRT-PCR using TaqMan Universal Master Mix II (no UNG, Life Technologies, South San Francisco, CA) and Taqman primers (Supplemental Table 3). All reactions were performed in triplicate for 40 cycles. We calculated the relative levels of expression between cases and controls using the ΔΔCT method.^24^ For each sample, mean cycle threshold (CT) values of each target gene were normalized to the mean CT value of glyceraldehyde-3-phosphate dehydrogenase (*GAPDH*), a commonly-used housekeeping gene.^25^ The levels of *GAPDH* expression were not significantly different between cases and controls (*P*>.05) in qRT-PCR (or RNA-seq analyses). Normalized values were adjusted to the average expression level of term controls. We determined significant differences in expression between cases and controls using an unpaired *t* test and a cutoff of *P*≤.05.

### Additional analyses

We interrogated the expression of DE genes between PTB and term cases using a publicly available, single-cell transcriptomic data set of the early gestation maternal-fetal interface.^26^ Normalized counts (ie, protein-coding transcripts per million [pTPM]) were obtained (E-MTAB-6701) and aligned with DE genes. Expression was evaluated across 18 cell types, for example, endothelial cells, STBs, and subsets of villous CTBs, extravillous CTBs, fibroblasts, and Hofbauer cells. We performed K-means cluster analysis (10 clusters, Euclidean Distance) to identify expression patterns that were specific to each cell type or subtype. Clusters of genes were evaluated for overrepresentation of GO terms (biological processes) using DAVID, and the 5 most highly enriched terms (by *P* value) were reported.

We also compared the DE transcripts we identified with data sets from other studies with similar designs to identify commonalities among the DE genes. The latter included (1) an analysis of placentas from PTB (n=20) and term births (n=13) by the Translational Health Sciences Institute of India that was performed in parallel with our study; (2) a recent profiling study by the ECHO Prenatal and Early Childhood Pathways to Health (PATHWAYS) consortium of 48 preterm and 540 term placentas^27^; and a meta-analysis^28^ that included 3 published transcriptomic data sets (GSE98224, GSE18809, GSE73685).^29^, ^30^, ^31^ The transcriptomic results from this study were combined with the aforementioned published data sets using the official gene symbol. Similar to Sobhani et al,^28^ the data sets were independently normalized, and gene expression was compared with that of the term samples in each group to examine the correlations among DE genes across gestational age.

## Results

### Morphologic analyses

#### Histopathology

The UCSF received placental biopsies from each of the 5 MOMI consortium collection sites. All sites used early ultrasound dating (<20 weeks’ gestation) to establish gestational age. PTB cases were defined as singleton deliveries at ≤36 weeks and 6 days completed gestation. Controls were defined as singleton deliveries at ≥37 weeks and 0 days completed gestation. The numbers were as follows: from AMANHI Bangladesh, there were 76 samples (38 PTB and 38 term); from GAPPS Bangladesh, there were 80 (35 PTB and 45 term) samples; AMANHI Pakistan contributed 80 (40 PTB and 40 term); AMANHI Tanzania contributed 45 (24 PTB and 21 term); and GAPPS Zambia contributed 60 (29 PTB and 31 term). The differences among the sites in the number of samples contributed were because of technical problems encountered in sectioning the blocks and the exclusion of HIV^+^ cases. The subject data for the participants are summarized in Supplemental Table 1.

**Figure 1:**
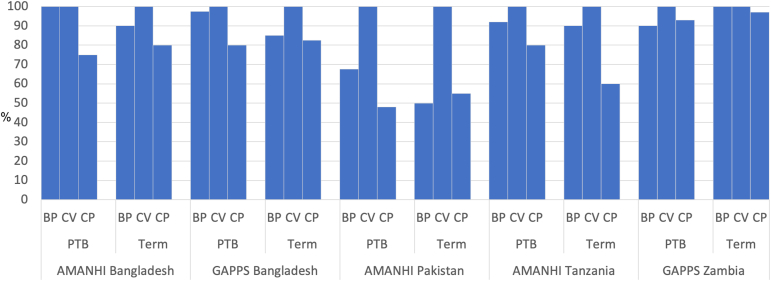
Composition of full thickness placental biopsies The bars indicate the percentage of biopsies that contained the basal plate (BP), chorionic villi (CV) or the chorionic plate (CP). The results are grouped by outcome (preterm birth [PTB] or term delivery). *AMANHI*, The Alliance for Maternal and Newborn Health Improvement; *BP*, basal plate; *CP*, chorionic plate; *CV*, chorionic villi; *GAPPS*, The Global Alliance to Prevent Prematurity and Stillbirth; *MOMI*, Multi-Omics for Mothers and Infants; *PTB*, preterm birth. *Robinson. Inflammationamong placentas from Multi-Omics for Mothers and Infants sites. Am J Obstet Gynecol 2025.*

The composition of the full thickness biopsies—that is, whether they contained the BP, CV of the placenta, and the CP—is illustrated in Figure 1 for each site and by outcome (preterm or term birth). All the biopsies contained CV. Whether or not the BP was sampled was more variable. The CP was the compartment that was most likely to be absent.

Initially, we scored tissue sections cut from the biopsies for a standard set of pathologic features (see Materials and Methods). From this preliminary analysis, we concluded that the amount of regional variability in many of the categories, evident in individual tissue sections, was too great to make firm conclusions. The exception was unmistakable signs of inflammation in terms of numbers and types of immune cells that were typically few in the BP, CV, and CP biopsies from uncomplicated term births in the United States. The percentage of cases with unequivocal evidence of inflammation, likely chronic villitis, was scored for each of these compartments.

**Figure 2:**
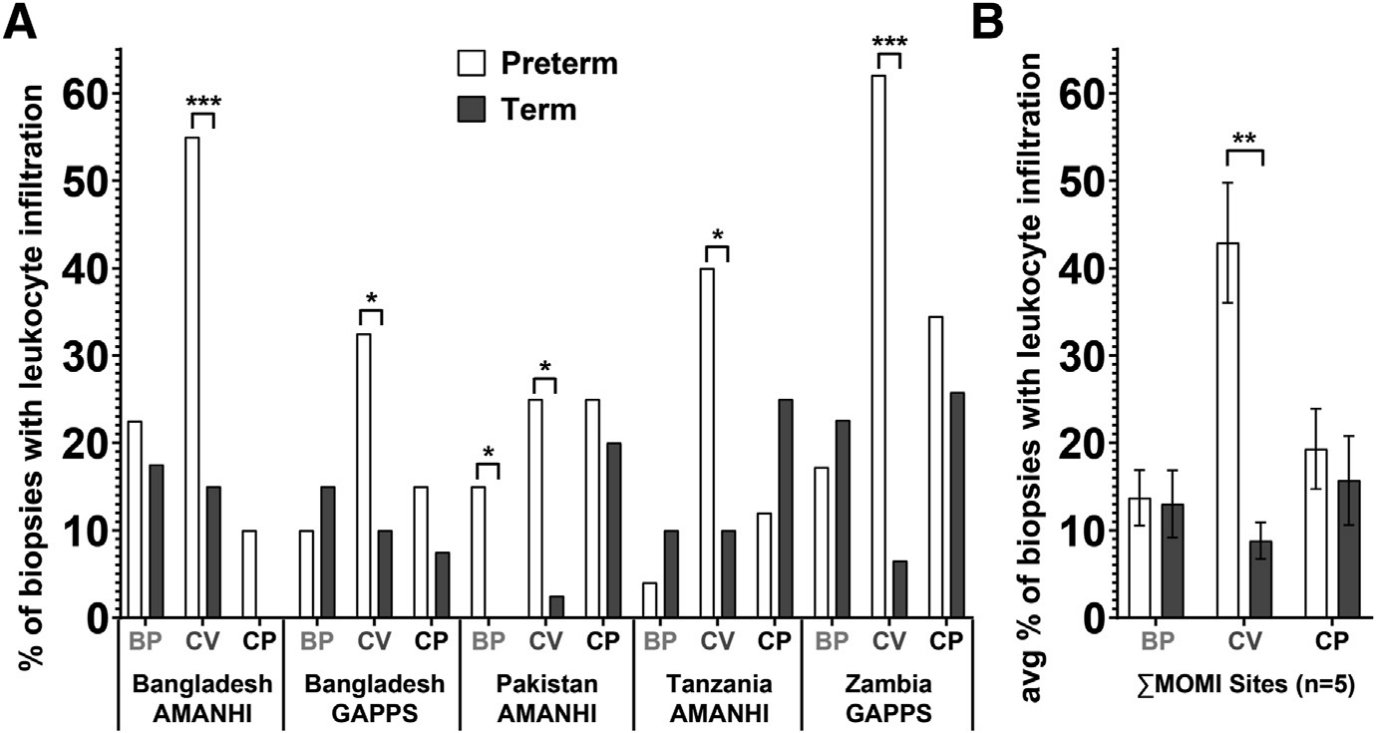
Microscopic evidence of increased inflammation in the placental chorionic villus compartment The bars indicate the percentage of biopsies that had evidence of leukocyte infiltration in either the basal plate (BP), chorionic villi (CV), or the chorionic plate (CP). The results are shown for the individual sites (**A**) and the consortium as a whole (**B**). The white bars indicate preterm births (<37 weeks’ gestation); the black bars indicate term births (≥37 weeks’ gestation). The asterisks denote significant differences between PTB and term births, determined using Fisher’s exact tests (panel **A**) or unpaired *t* tests (Panel **B**). *Asterisk* indicates *P*<.05; *double asterisks* indicate *P*<.005; and *triple asterisks* indicate *P*<.0005. *AMANHI*, The Alliance for Maternal and Newborn Health Improvement; *BP*, basal plate; *CP*, chorionic plate; *CV*, chorionic villi; *GAPPS*, The Global Alliance to Prevent Prematurity and Stillbirth; *MOMI*, Multi-Omics for Mothers and Infants; *PTB*, preterm birth. *Robinson. Inflammation among placentas from Multi-Omics for Mothers and Infants sites. Am J Obstet Gynecol 2025.*

Figure 2 summarizes these data (average percentage of biopsies with leukocyte infiltration) for each MOMI site (panel A) and for the entire consortium (panel B). The most commonly affected compartment was the CV in PTB cases, which ranged from 25% at the Pakistan site to 62% at the Zambia site (panel A). A comparison of the data from the 2 Bangladesh cohorts suggested that the AMANHI site (55%) had a higher incidence than the GAPPS cohort (33%). The proportion of CV with leukocyte infiltration in PTB vs term samples was significantly higher at all 5 collection sites (*P*<.05). Across the consortium, 42% of the CV samples from PTB cases had histologic evidence of inflammation compared with 9% of the control group (panel B; *P*<.005). In addition to findings that were generalizable, particular features were specific to certain biorepositories. For example, the percentage of biopsies from the Pakistan site with evidence of CP inflammation in both the preterm and term groups approximated the inflammation in the CV in PTB cases and was significantly different when compared to term (*P*<.05). Involvement of this region may be a sign of ascending infection,^32^ which negatively affected pregnancy outcomes only in the PTB group. Unless noted above, lower levels of inflammation, which were not statistically significant and did not correlate with case or control status, were observed in association with the other compartments.

**Figure 3:**
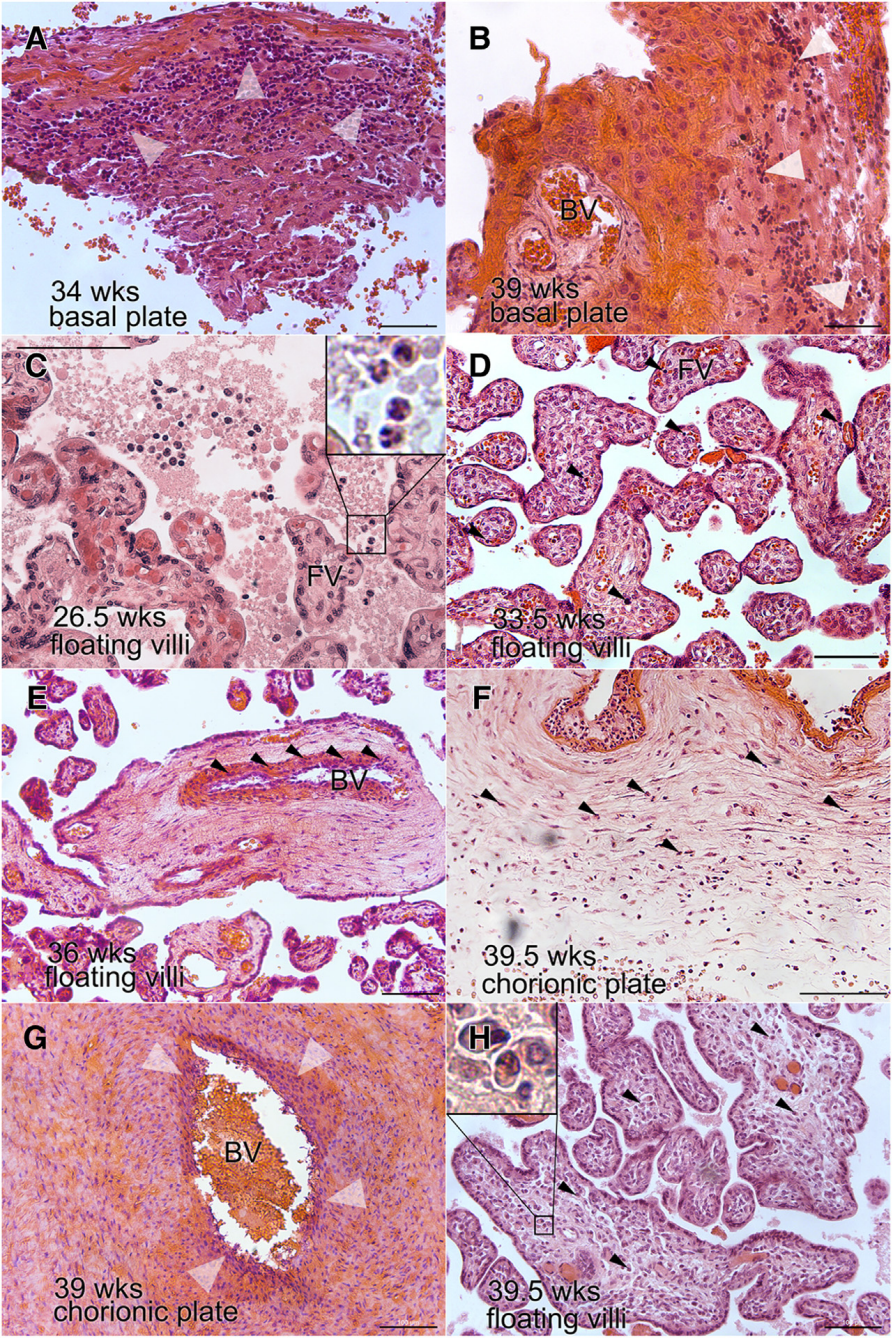
Examples of inflammatory lesions observed in the placental biopsies **A,** Basal plate: at the maternal-fetal interface, robust leukocyte infiltration (arrowheads) of the decidua was the most frequent finding. **B,** In some cases, the perivascular regions of uterine blood vessels (BVs) were infiltrated along with the decidua (arrowheads). Chorionic villi: leukocyte infiltration was most frequently associated with chorionic villi from the preterm group (Figure 2). **C,** An example of cells with a neutrophil morphology in the intervillous space (see inset). **D,** Hofbauer cell (fetal macrophage; arrowheads) infiltration of the villus stroma in a PTB case. **E,** Leukocyte infiltration (arrowheads) of a BV and its walls within the villus stroma. **F,** Chorionic plate: leukocyte infiltration was a relatively common feature (arrowheads), (**G**) which sometimes involved the BVs in this region (arrowheads). **H,** Rare pathologic features: in this example, there was abundant plasma cell infiltrate (arrowheads) of the chorionic villous stroma (see inset). Bars = 100 μm. *BV*, blood vessel; *FV*, floating villi; *PTB*, preterm birth. *Robinson. Inflammation among placentas from Multi-Omics for Mothers and Infants sites. Am J Obstet Gynecol 2025.*

Figure 3 illustrates the most common types of inflammatory lesions that were observed in the BP, CV, and CP. With regards to the BP, robust leukocyte infiltration of the decidua was the most frequent finding (Figure 3, A). In some cases, the pericellular regions associated with uterine blood vessels (BVs) were similarly affected (Figure 3, B). Analysis of the placental CV showed that signs of inflammation were most frequently observed in the PTB groups across all the sites (Figure 2). Leukocytes, including neutrophils (Figure 3, C), were evident in the intervillous spaces. Hofbauer cell (macrophage) hyperplasia was also observed (Figure 3, D). Leukocyte infiltration of the BVs and their walls within the villus stroma was another prominent feature (Figure 3, E). As for the CP, leukocyte infiltration, presumably involving fetal cells, was a relatively common feature (Figure 3, F), which sometimes involved the BVs in this region (Figure 3, G). We also found isolated features in individual specimens. As shown in Figure 3, H, this included a term birth in which we observed abundant plasma cells infiltrating the chorionic villous stroma.

### Transcriptomics

**Figure 4:**
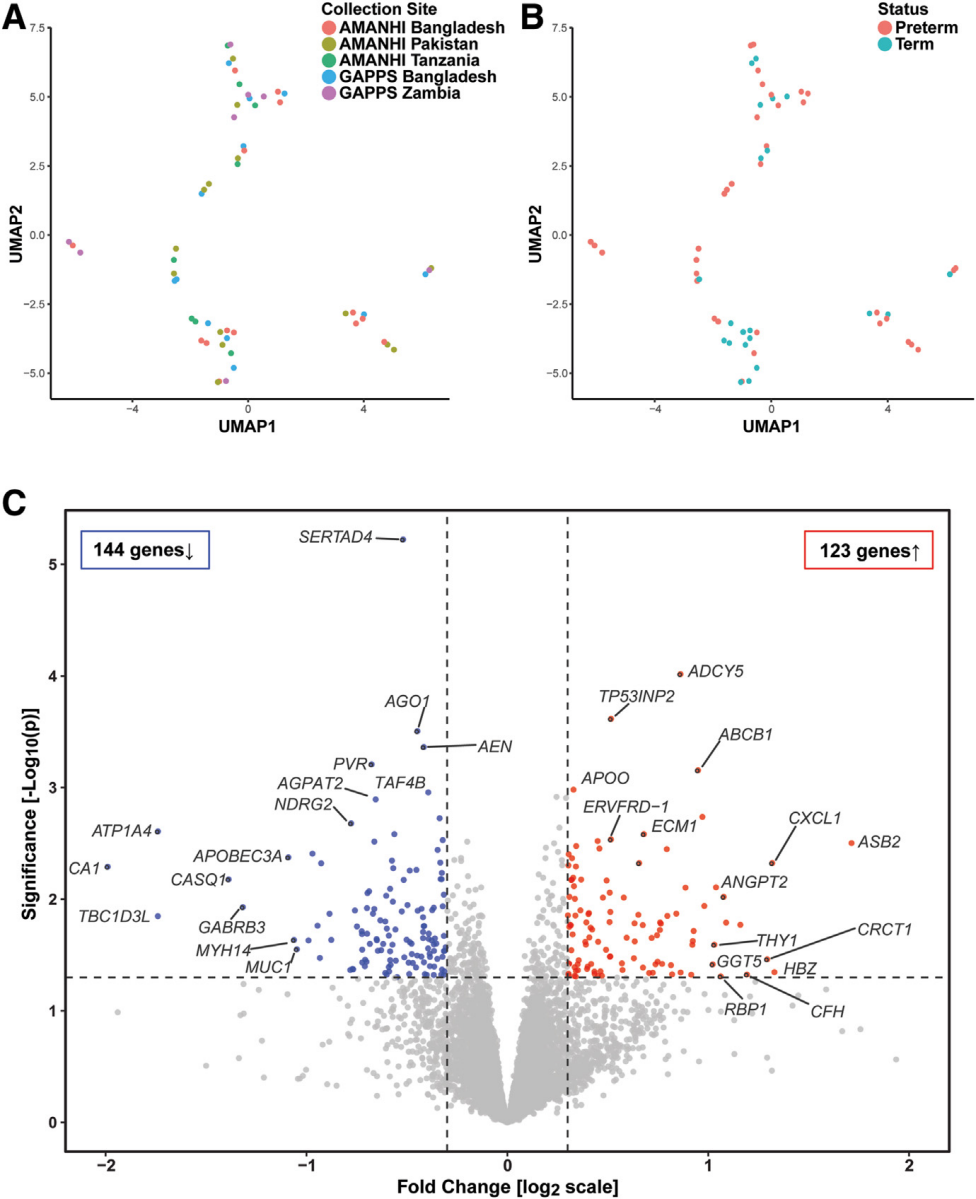
Global gene expression analysis of placentas from preterm and term births revealed DE genes Uniform Manifold Approximation and Projection (UMAP) analysis showed that samples failed to cluster according to (**A**) collection site or (**B**) gestational age at birth. **C,** Volcano plot displaying significance (y-axis) and mean fold change (FC) (x-axis) differences in gene expression between preterm and term chorionic villi. Red indicates up-regulated genes (n=123) and blue indicates down-regulated genes (n=144). *AMANHI*, The Alliance for Maternal and Newborn Health Improvement; DE, differentially expressed; *FC*, fold change; *GAPPS*, The Global Alliance to Prevent Prematurity and Stillbirth; *UMAP*, Uniform Manifold Approximation and Projection. *Robinson. Inflammation among placentas from Multi-Omics for Mothers and Infants sites. Am J Obstet Gynecol 2025.*

RNA from 56 of the placentas were subject to histopathology analyses. The number of samples from each of the 5 MOMI sites was as follows: AMANHI Bangladesh, 14 (10 PTB and 4 term); GAPPS Bangladesh, 12 (4 PTB and 8 term); AMANHI Pakistan, 14 (7 PTB and 7 term); AMANHI Tanzania, 8 (8 PTB); and GAPPS Zambia, 8 (6 PTB and 2 term). Differences among the sites in the number of samples contributed were because of exclusion of those with a RIN <7. The subject data for the participants is summarized in Supplemental Table 2. The UMAP analysis showed that the data did not cluster according to the MOMI collection site (Figure 4, A) or gestational age at time of delivery (Figure 4, B).

We identified 267 genes that were DE between placentas from PTB pregnancies and those from term pregnancies (uncorrected *P*<.05; absolute fold change [FC] ≥1.25) (Figure 4, C). Of this subset, 123 were upregulated (red) and 144 were downregulated (blue) in case vs control samples. The most highly upregulated genes included: *ASB2, CXCL1,* cysteine-rich C-terminal protein 1 (*CRCT1*), complement factor H (CFH), *ANGPT2,* retinol binding protein 1 (*RBP1*), THY-1 cell surface antigen (*THY1*), and gamma-glutamyltransferase 5 (*GGT5*). The down-regulated gene with the highest statistical significance was *SERTAD4* (*P*=.000006). The others (uncorrected *P*<.05; absolute FC ≥1.25) included: poliovirus receptor (PVR, CD155), *CA1,* ATPase Na+/K+ transporting subunit alpha 4 (*ATP1A4*), calsequestrin 1 (*CASQ1*), gamma-aminobutyric acid type A receptor subunit beta 3 (*GABRB3*), apolipoprotein B messenger RNA editing enzyme catalytic subunit 3A (*APOBEC3A*), myosin heavy chain 14 (*MYH14*), *and* mucin 1 (*MUC1*) (cell surface associated).

Individual genes had interesting functions that could be relevant to PTB. The placental roles of *SERTAD4* are not known. However, in other contexts, it functions as a transcriptional cofactor.^33^ This gene is highly expressed in trophoblasts and were identified in a recent meta-analysis of PTB data sets.^28^ *ASB2* encodes a ubiquitin ligase subunit critical for cardiomyocyte differentiation.^34^ Whether this molecule plays a similar role in trophoblasts is unknown. *APOBEC3A* restricts transmission of foreign DNA, such as from viruses.^35^ *PVR* has roles in adhesion, invasion, and immune defense.^36^

**Figure 5:**
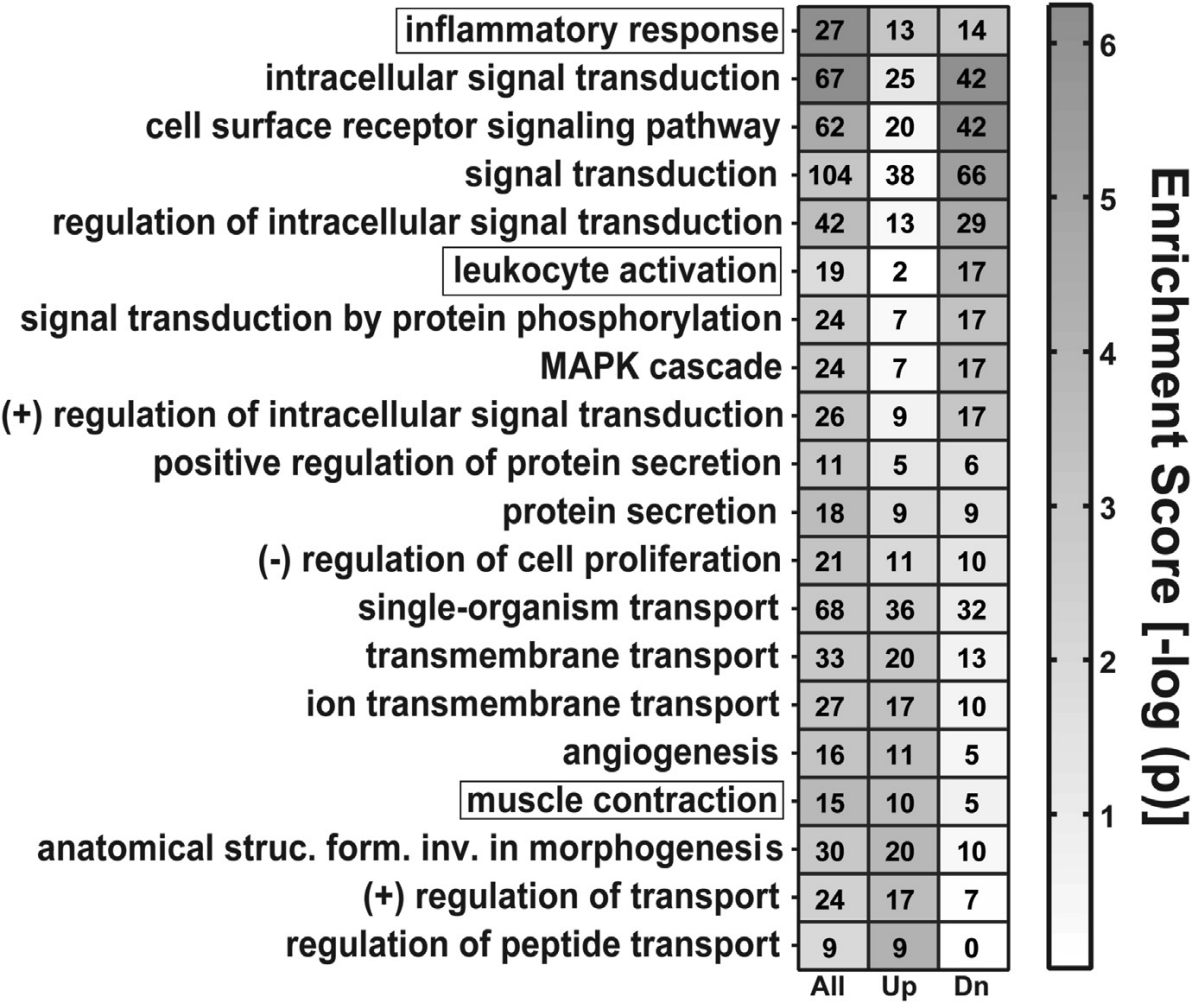
Mapping of DE genes to GO biological processes In total, 20 GO terms were enriched in genes that were DE between preterm and term chorionic villi (*P*<.001; n≥8 DE genes per pathway). The total number of genes (All), as well as those that were up-regulated (Up) and down-regulated (Dn), is shown. Shading denotes significance (−log [*P*]). *DE*, differentially expressed; *GO*, gene ontology. *Robinson. Inflammation among placentas from Multi-Omics for Mothers and Infants sites. Am J Obstet Gynecol 2025.*

The GO analysis identified 20 biologic processes as enriched in samples from the PTB cases (Figure 5). Consistent with the histologic analyses that frequently detected signs of inflammation, leukocyte activation and inflammatory responses were among the highlighted processes, as well as muscle contraction, a key component of birth. Many aspects of transport (ie, peptides, ions, and single organisms) and signaling (eg, transduction, via cell surface receptors; intracellular; and the MAPK cascade) were impacted. Other affected processes included angiogenesis, morphogenesis, protein secretion, and negative regulation of cell proliferation. All but one of the categories (peptide transport) identified comprised up- and down-regulated genes, suggesting the important role of balanced expression in normal pregnancy and imbalances in PTB.

**Supplemental Figure 1:**
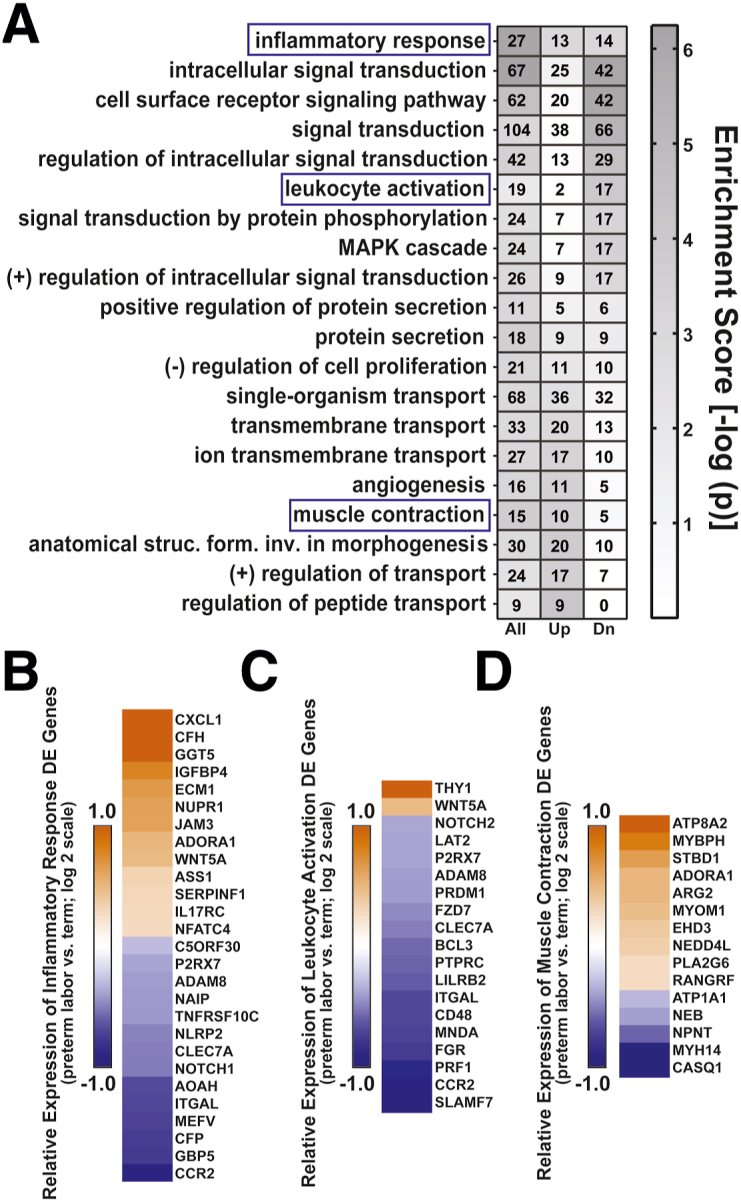
DE genes in selected GO pathways **A,** GO pathways replicated from Figure 5. **B,** Inflammatory response pathway. **C,** Leukocyte activation. **D,** Muscle contraction. *DE*, differentially expressed; *GO*, gene ontology. *Robinson. Inflammation among placentas from Multi-Omics for Mothers and Infants sites. Am J Obstet Gynecol 2025.*

Exploration of gene functions associated with enriched biologic processes further emphasized the diversity of dysregulated molecules that correlated with PTB (Supplemental Figure 1). For example, up-regulated genes that were linked with the inflammatory response GO term included *CXCL1*, an antimicrobial cytokine and robust biomarker of lipopolysaccharide-induced PTL in mice,^37^ and *CFH*, a previously identified biomarker of idiopathic, spontaneous PTL in humans.^38^ Genes associated with leukocyte activation and down-regulated in PTB included C-C chemokine receptor type 2 (*CCR2*), a chemokine that mediates monocyte chemotaxis,^39^ and cluster of differentiation 48 (*CD48*), a B-lymphocyte activation marker.^40^ Together, the pattern of gene regulation in these pathways suggested the inhibition of leukocyte activation in the context of inflammation. Dysregulated genes associated with muscle contraction included myomesin-1 (*MYOM1*), a filament protein implicated in the regulation of contractility and myocardial atrophy,^41^ and *CASQ1*, a binding protein involved in the regulation of calcium homeostasis.^42^

**Figure 6:**
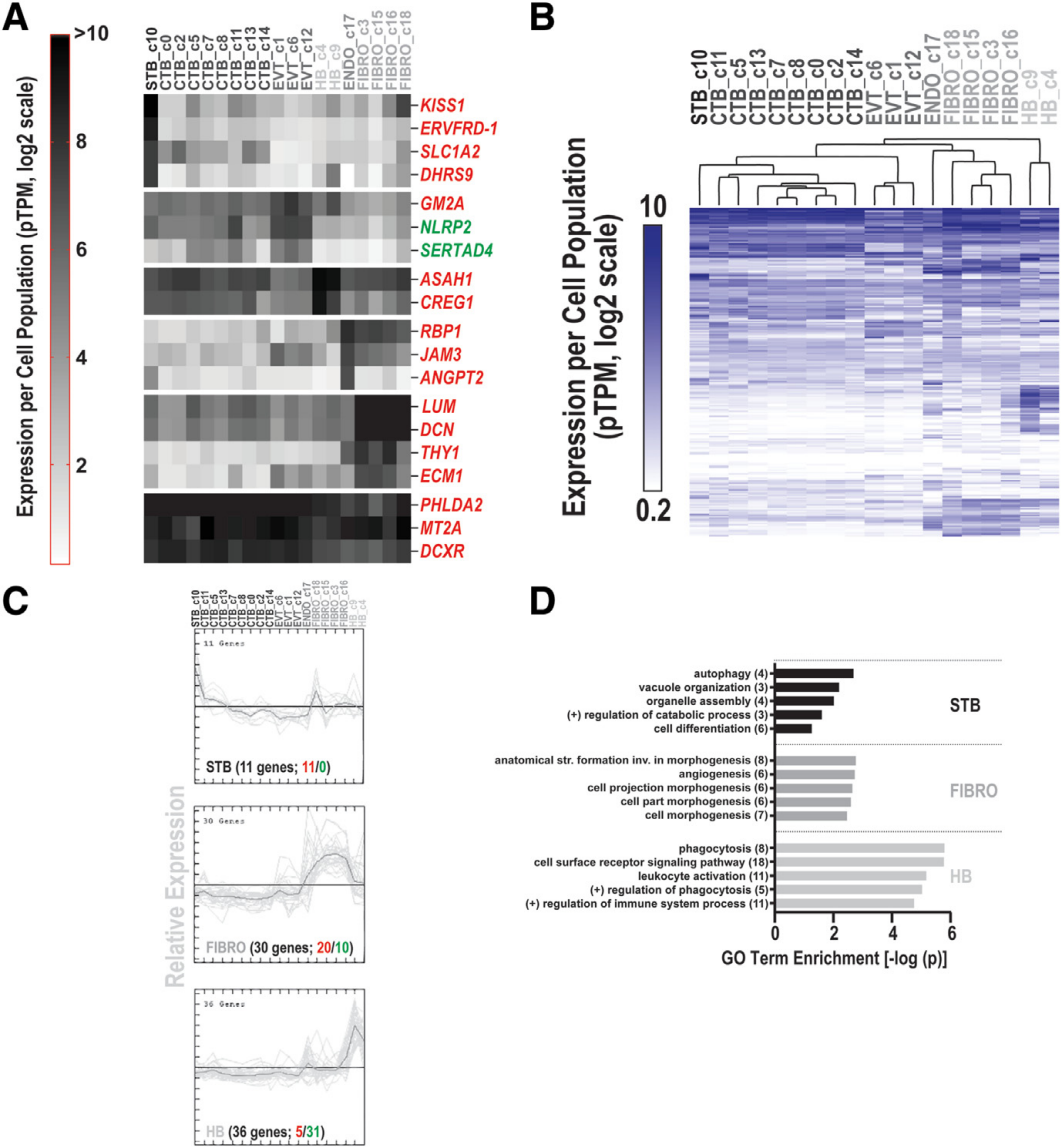
Single-cell transcriptomic profiles of DE genes **A,** Examples of placental genes with cell type– and subtype-specific expression patterns. Genes are color coded based on predicted dysregulation in PTB vs term (up-regulated, red; down-regulated green). **B,** Hierarchical clustering of the DE genes revealed cell type specificity. **C,** K-means clustering analysis identified clusters that were specific to syncytiotrophoblasts (STBs) (n=11), fibroblasts (FIBRO) (n=30), or Hofbauer cells (HB) (n=36). **D,** Shows the GO biologic process enrichment analysis of the population-specific clusters. The 5 most significantly enriched terms are shown for each cluster. *CTB*, cytotrophoblast; *DE*, differentially expressed; *Endo*, endothelial cells; *EVT*, extravillous trophoblast; *FIBRO*, fibroblasts; *HB*, Hofbauer cells; *PTB*, preterm birth; *STB*, syncytiotrophoblast. *Robinson. Inflammation among placentas from Multi-Omics for Mothers and Infants sites. Am J Obstet Gynecol 2025.*

Next, we mapped the data onto a recent single-cell transcriptomic study of the maternal-fetal interface.^26^ The goal was to identify the cell types that were likely involved in the dysregulated expression of specific genes (Figure 6, A). The heat map shows a portion of the published gene expression data for the DE genes from this study, which are labeled on the right (red, up-regulated; green, down-regulated). Several patterns emerged. Some genes were primarily expressed by STBs (kiss-1 metastasis suppressor [*KISS1*], endogenous retrovirus group FRD member 1, envelope [*ERVFRD-1*]*,* solute carrier family 1 member 2 [*SLC1A2*], and dehydrogenase/reductase 9 [*DHRS9*]). Transcripts for others were present at higher levels in trophoblasts than in most other placental cell types (GM2 ganglioside activator [*GM2A*]*,* NLR family pyrin domain containing [*NLRP2*] and *SERTAD4*). Some gene products were more abundant in nontrophoblast cell types. Hofbauer cells contained N-acylsphingosine amidohydrolase 1 (*ASAH1*) and cellular repressor of E1A stimulated genes (*CREG1*); endothelial cells contained *RBP1,* junctional adhesion molecule 3 (*JAM3*) and *ANGPT2*; and fibroblasts contained lumican (*LUM*)*,* decorin (*DCN*)*, THY1*, and extracellular matrix protein 1 (*ECM1*). Lastly, a subset was highly expressed in all the annotated placental cell types, including pleckstrin homology like domain family A member 2 (*PHLDA2*)*,* metallothionein 2A (*MT2A*), and dicarbonyl and L-xylulose reductase (*DCXR*). Together, these results suggest that PTB impacts gene expression in trophoblast and nontrophoblast cells of the placenta. More broadly, we concluded that this strategy can be used to map DE genes from bulk RNA-seq experiments to specific cell types of the placenta or any organ or tissue for which single cell RNA-seq data are available.

To gain insights into how PTB could differentially affect the functions of placental components, we performed GO enrichment analysis of subpopulation-specific DE genes. As expected, hierarchical clustering separated the data by cell type and subtype (Figure 6, B). K-means clustering enabled stratification of the genes that were DE in PTB based on their cell type or subtype-specific expression patterns (Figure 6, C). The results identified 3 significant clusters (>10 genes) specific to STBs (n=11), fibroblasts (n=30), or Hofbauer cells (n=36). They were enriched for genes with diverse functions. For example, STBs were enriched for genes critical to autophagy, fibroblasts were enriched for genes that were involved in morphogenesis and angiogenesis, and Hofbauer cells (placental macrophages) were enriched for genes associated with phagocytosis and leukocyte activation (Figure 6, D). Taken together, these data suggested that PTB had specific effects on the transcriptomes of these placental cell subpopulations in the context of other genes whose placental expression was more broadly impacted.

**Figure 7:**
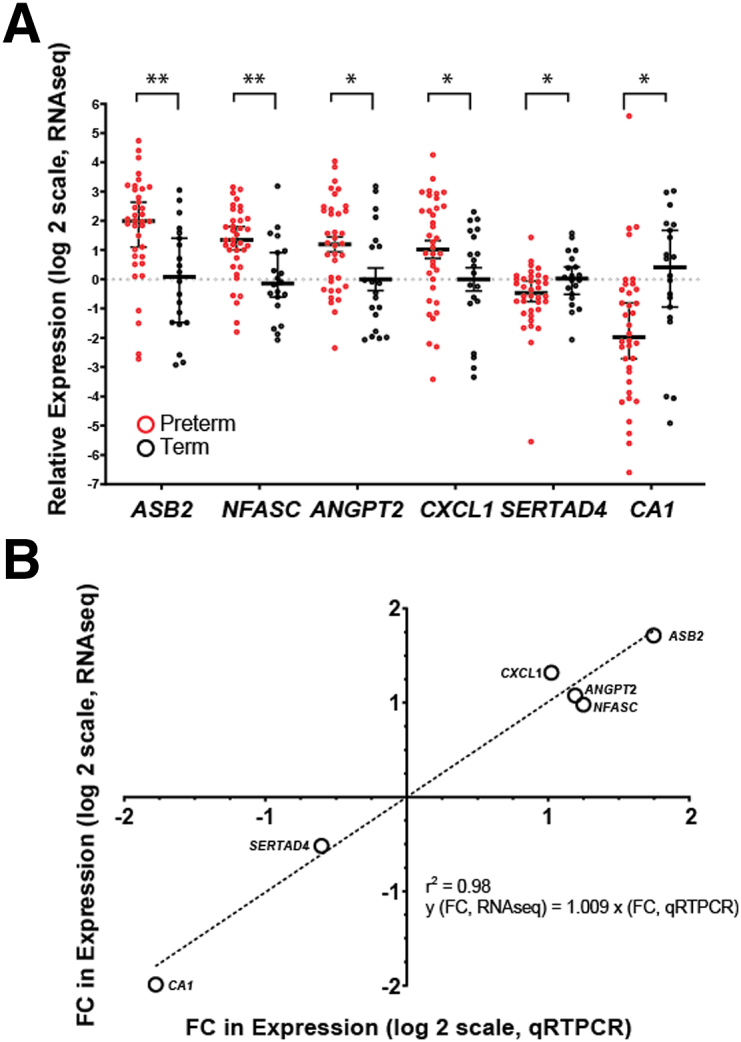
qRT-PCR validation of selected targets **A,** Confirmation of targets in PTB cases (n=36) vs term controls (n=20) via qRT-PCR. The asterisks represent significant differences between PTB and term controls (*t* test; *asterisk* indicates *P*<.05; *double asterisks* indicate *P*<.005). **B,** Correlation between average fold change (FC) values for candidate genes derived using qRT-PCR and those derived using RNA-seq (R^2^=.98). *FC*, fold change; *PTB*, preterm birth; *qRT-PCR*, quantitative reverse transcriptase–polymerase chain reaction; *RNA-seq*, RNA sequencing. *Robinson. Inflammation among placentas from Multi-Omics for Mothers and Infants sites. Am J Obstet Gynecol 2025.*

Finally, we validated 6 DE genes between cases and controls using qRT-PCR expression (Figure 7). For these analyses, we selected 4 genes that were up-regulated in PTB (*ASB2*, *NFASC*, *ANGPT2, CXCL1*) and 2 genes that were down-regulated in PTB (*SERTAD4*, *CA1*). Significant differences in expression between PTB and controls were confirmed for all 6 (Figure 7, A). The average FC differences between PTB cases snd term controls, quantified using qRT-PCR, were similar to the FC differences obtained via RNA-seq (R^2^=.98) (Figure 7, B). Our analysis highlights specific molecules that could serve as candidate placental biomarkers and therapeutic targets in the setting of PTB.

## Comment

### Principal findings

The histopathology data from this study suggested a high prevalence of inflammation at baseline of the BPs, placenta or CV, and CPs among the samples donated by the MOMI participants. The actual rates are almost certainly higher. The fetal membranes and umbilical cords, frequently examined for signs of inflammation or infection, were not collected by all MOMI biorepository sites. Furthermore, our analysis was confined to 1 full thickness placental biopsy from each case, and we scored only unequivocal signs of inflammation, such as extensive leukocyte infiltration and the other examples shown in Figure 3. The fact that, taken as a group, all 3 compartments were affected suggested that ascending infection, hematogenous routes, and/or a maternal immunologic response to the fetus are important contributors to the observed inflammation. Despite high rates in all the compartments, only inflammation involving the CV, likely chronic villitis, correlated with PTB. The large number of samples we analyzed made the latter observation a highly important finding. The transcriptomic analyses, which involved fewer but still substantial numbers of samples, also identified inflammation-related genes and pathways as being up-regulated in PTB. Our findings are in line with a previous histologic analysis of pregnant women with an HIV infection in Uganda that found a high rate of chorioamnionitis that correlated with adverse birth outcomes.^43^ In contrast, lower rates of chronic intrauterine infections (20.8%) were reported in a Korean study of late PTBs,^44^ which the majority of our cases were. We theorize that it is possible that underlying undiagnosed infections (other than HIV, which we excluded) could contribute to the high rates of inflammation we observed.

### Results in the context of what is known

The complex pathogenesis of PTB remains poorly understood, which is a large contributor to the limited availability of targeted and effective strategies for prevention and treatment of PTB.^45^ There are several well-recognized risk factors for this pregnancy complication that were summarized in the Introduction.^46^, ^47^, ^48^, ^49^ However, a large proportion of PTBs have no identifiable cause. These spontaneous cases present as pathologic inflammation, preterm premature rupture of the membranes (PPROM), and the onset of PTL.^50^ Inflammation is also a key physiological trigger of normal labor at term.^45^^,^^51^ Although the role of the maternal immune system is well recognized, recent work also points to the fact that fetal inflammation in response to maternal antigens can induce uterine contractions and PTL. Thus, in some cases, the fetus may be an instigator rather than a passive bystander.^52^

An unbiased analysis suggested that microbial invasion of the fetal membranes and colonization of amniotic fluid was more common than expected in PPROM.^53^ Accumulating evidence suggests that microorganism-induced metabolic dysregulation at the maternal-fetal interface stimulates production of inflammatory cytokines that may initiate PTB.^54^ Components of the uterine microbiome including bacteria—and to a lesser extent viruses and fungi—are associated with negative pregnancy outcomes including PTB. The most significant associations are with *Ureaplasma* spp. and *Fusobacterium* spp.^55^ Furthermore, *Listeria monocytogenes*, a common contaminant of a variety of raw foods, can infect the placenta and lead to PTB.^56^ The parasite *Toxoplasma gondii*, spread by food and domestic animal excrement, can also breach the placental barrier and cause adverse pregnancy outcomes.^57^ Recent data suggest that, under normal circumstances, bacteria can colonize the gastrointestinal tract before birth,^58^ raising the possibility that infectious processes could illicit a fetal immune response. Identifying the sites of infection and the biologic mechanisms that control microbe-host interactions is a difficult but essential endeavor for improving PTB prevention and treatments. In this regard, our study described many possible routes of infection and the types of immune cells that are deployed in response.

**Supplemental Figure 2:**
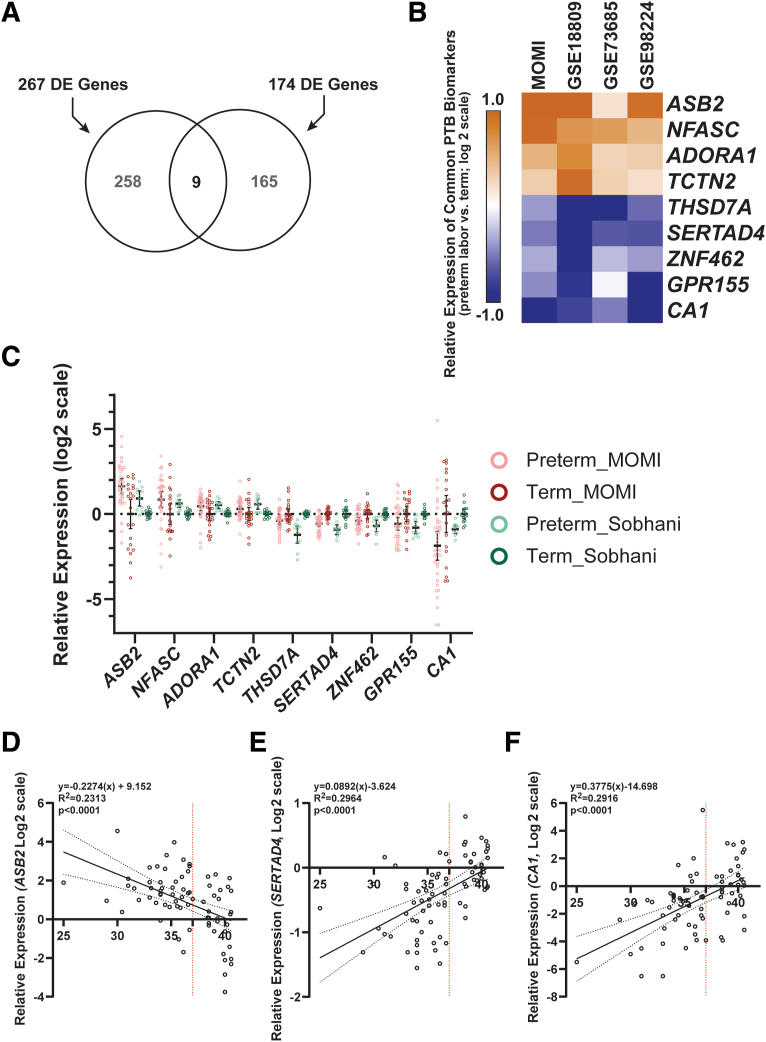
The MOMI data vs Sobhani et al,^28^ 2022 **A,** A meta-analysis of 3 published placental transcriptomic data sets (total PTB cases, n=11 vs total term controls, n=15) identified 174 genes that were DE in cases vs controls. An aggregated analysis that included the MOMI data (267 DE genes) revealed that 9 genes were common in all 4 studies. **B,** As shown in the heatmap, they were also regulated in the same manner. **C,** Shows the gene expression levels of individual samples in comparison with their average control values in the Sobhani meta-analysis and the MOMI data set. Plotting of the data in panel **C** showed that expression of (**D**) *ASB2*, (**E**) *SERTAD4*, and (**F**) *CA1* significantly correlated (*P*<.0001) with gestational age at birth. *DE*, differentially expressed; *MOMI*, Multi-Omics for Mothers and Infants. *Robinson. Inflammation among placentas from Multi-Omics for Mothers and Infants sites. Am J Obstet Gynecol 2025.*

DE placental genes associated with PTB in this study were also identified in other transcriptomic placental studies with similar designs. Recently, we performed a meta-analysis of 3 published placental transcriptomic data sets (total PTB cases, n=11 vs total term controls, n=15) to identify DE genes that were shared across the studies.^28^ Although most DE genes were unique to individual studies, an aggregated analysis of these data sets identified a total of 174 genes that were DE in PTB vs term birth (Supplemental Figure 2, A). A comparison with the MOMI data set revealed that 9 of these genes were common to all 4 studies. A heatmap showed that they were regulated in the same manner (Supplemental Figure 2, B). *ASB2*, neurofascin (*NFASC*), adenosine A1 receptor (*ADORA1*), and tectonic family member 2 (*TCTN2*) were up-regulated. Thrombospondin type 1 domain–containing 7A (*THSD7A*), *SERTAD4*, zinc finger protein 462 (*ZNF462*), *G* protein-coupled receptor *155* (*GPR155*), and *CA1* were down-regulated. Plotting gene expression levels of individual samples in comparison with their average control values in the Sohbani meta-analysis and the MOMI data set showed a high degree of concordant expression.^28^ *ASB2* (Supplemental Figure 2, D), *SERTAD4* (Supplemental Figure 2, E), and *CA1* (Supplemental Figure 2, F) were significantly correlated (*P*<.0001) with gestational age at birth, suggesting they might warrant investigation for their roles in PTB and having potential use as biomarkers.

Subsequent to this meta-analysis, the ECHO PATHWAYS consortium conducted a transcriptomic study of 48 preterm and 540 term placentas. They discovered 1729 genes that were DE in spontaneous PTB.^27^ Of these genes, 53 were also DE in the MOMI data set (Supplemental Table 4), and 30 had significant associations with early PTB (<34 weeks of gestation). A majority (∼85%) of the common DE genes showed similar trends (fold changes) in both studies. Notably, several genes were up-regulated, including *ASB2*, arginase, type II (*ARG2*), and Unc-51 like kinase 4 (*ULK4*). Those that were down-regulated included *APOBEC3A*, CD300E molecule (*CD300E*), FGR proto-oncogene, Src family tyrosine kinase (*FGR*), complement factor properdin (*CFP*), myeloid cell nuclear differentiation antigen (*MNDA*), MEFV innate immunity regulator (*MEFV*), BCL2 related protein A1 (*BCL2A1*), *CD48*, vascular noninflammatory molecule 2 (*VNN2*), acyloxyacyl hydrolase (*AOAH*), leukocyte immunoglobulin-like receptor B3 (*LILRB3*), amine oxidase copper containing 1 (*AOC1*), *SERTAD4*, and notch receptor 1 (*NOTCH1*). These molecules may be important candidate biomarkers because of their association with early PTB and differential magnitude of expression in PTB vs term births (absolute fold change >0.5 in both studies).

A recent genome-wide meta-analysis of gestational duration (n=195,555) identified 22 loci (24 independent variants) and an enrichment of genes differentially expressed during labor.^59^ A meta-analysis of PTB (18,797 cases; 260,246 controls) revealed large genetic similarities with gestational duration. Fifteen of the gestational duration genetic variants acted through the maternal genome, 7 acted through both the maternal and fetal genomes, and 2 acted only through the fetal genomes. Messenger RNAs associated with 3 of these variants, transmitted as indicated, were DE in our study, namely *HIVEP3* (*P*=.005, maternal); *TET3* (*P*=.017, maternal and fetal); and *ADCY5* (*P*=.001, fetal). Coalescing transcriptomic data with genetic data sets could help to pinpoint the tissues and cell types that contribute to the pathogenesis of PTB.

In an analysis of PTB (n=20) and term (n=13) placentas by the Translational Health Sciences Institute of India that was conducted in parallel with our investigation (Kheterpal et al, unpublished data), 20 genes were identified as being DE (Bonferonni adjusted *P*<.05; absolute FC ≥1.5). Three genes in this subset—NDRG family member 2 (*NDRG2*), *CA1*, L1 cell adhesion molecule (*L1CAM*)*—*were also identified as being DE in the MOMI data set and followed similar trends (fold changes) when PTB was compared with term birth samples (R^2^=.88) (Supplemental Table 5). This result suggested a modest overlap in the number of DE genes between the 2 data sets but a high degree of consistency in those that overlapped.

### Clinical implications

As discussed previously, there is a great deal of evidence that inflammation is associated with PTB in developed countries. However, there is little information about this relationship in low- and middle-income countries. The data presented here suggest a positive association in low- and middle-income countries as well, strengthening the clinical importance of this finding, which could be more broadly generalizable than currently appreciated.

Although the use of antibiotic therapies as preventative and treatment strategies for women in developed countries have been controversial and largely unsuccessful (American College of Obstetricians and Gynecologists practice Bulletin No. 234),^60^^,^^61^ it may be that treating women in developing countries, who have had less exposure to these agents, might have benefits either before or after the onset of PTL. However, therapies targeted to causative agents yet to be determined will probably be required; more generally efficacious antibiotics were not effective in a prevention trial conducted in Bangladesh.^62^ In this regard, maternal dysbiosis transmitted to the fetus could prevent microbiome maturation in the offspring, leading to subsequent impaired growth, another setting in which antibiotic treatment of women in developing countries might be efficacious.^63^ Finally, the route of antibiotic delivery is also important. Antibiotic administration can eradicate intraamniotic infection or intraamniotic inflammation in a subset of patients with PTL and intact fetal membranes.^64^

### Research implications

In summary, we found a high prevalence, at baseline, of inflammation in all the compartments of the full thickness placental biopsies that were collected by the MOMI consortium. Even higher rates were observed in the placental CV from pregnancies complicated by PTB. This finding raises several questions that will be important to answer. Is there evidence of dysbiosis before pregnancy? If so, what are the major microbial drivers and are they amenable to eradication and establishment of a maternal environment that is more conducive to a normal pregnancy?

### Strengths and limitations

A strength of this study was the analysis of specimens collected from geographical locations in which very little is known about the placenta in the context of normal labor at term birth or PTB. Furthermore, it was informative to compare the rates of inflammation among the 5 MOMI biorepository sites in Africa and southeast Asia. In this regard, there were differences between the 2 Bangladesh sites, suggesting societal and environmental factors that differ between the 2 areas of the same country. In addition, when compared with other published studies, we analyzed a substantial number of samples.

The limitations include that our analyses were confined to a single biopsy per workflow. Thus, we scored samples with evidence of inflammation in a single area as positive, which constituted only a small portion of the maternal-fetal interface and placenta. In addition, the fetal membranes and umbilical cords were not collected by the sites and thus were not analyzed in this study. Inevitably, these combined factors led to an underestimation of the incidence of inflammation. In addition, only a portion of the biopsies contained all 3 regions of interest—the BP, CV, and CP. Furthermore, because the controls were from term placentas, we could not rule out the possibility that the inflammation we observed was a normal feature of the earlier gestational age of the preterm samples. The same caveats apply to our RNA-seq analysis; 1 biopsy is only a window into the placenta as a whole. In addition, the Bangladesh data cautioned against extrapolation of the findings to other areas of the same country or global region because site-specific factors may be playing an important role in the rates of placental inflammation. Finally, because the biorepositories contributed very different numbers of samples as a whole and from term and preterm deliveries, we could not analyze the RNA-seq data by site.

### Conclusion

Our data point to a much higher than expected incidence of inflammation at the maternal-fetal interface in preterm and term placental samples collected by the MOMI biorepository sites. This finding raises the possibility that pregnancies in certain developing regions around the world may be affected similarly. Our results warrant further investigation. It will be important to determine how widespread this phenomenon is in the relevant geographies. Among pregnancies that continue to term, it is possible that there are important associations with other adverse outcomes, such low birth weight. In pregnancies that ended in a PTB, further exploration of the causes and possible remedies is warranted.

## Figures and Tables

**Supplemental Table 1:** Subject data for the participants whose placentas were used in the histopathology analyses

Collection site	Subset	n	Maternal age (y)	Gestational age (wk)	Birth weight (g)	Inductions	Cesarean deliveries	Baby sex	Maternal education (y)
			Mean (SD)	Mean (SD)	Mean (SD)	#	#		Mean (SD)
AMANHI Bangladesh	Total	76	23.7 (4.5)	36.4 (3.7)	2511.5 (493)	0	0	30 M, 46 F	5.3 (3.6)
	Term	38	24.6 (4.4)	39.3 (1.3)	2782.4 (347.3)			16 M, 22 F	6.4 (2.4)
	PTB	38	22.7 (4.5)^a^	33.6 (3.0)^b^	2168.3 (435.4)^b^			14 M, 24 F	4.1 (4.2)^a^
AMANHI Pakistan	Total	80	25.7 (5.1)	37.2 (2.5)	2480.6 (499.5)	2	2	40 M, 40 F	7.0 (4.1)
	Term	40	25.7 (5.3)	39.3 (1.2)	2792.6 (321.0)	0	0	22 M, 20 F	6.3 (4.1)
	PTB	40	25.6 (4.9)^a^	35.0 (1.4)^b^	2125.4 (424.5)^b^	2	2	18 M, 20 F	7.8 (4.1)^a^
AMANHI Tanzania	Total	45	28.1 (6.0)	36.9 (3.3)	3062.0 (685.8)	2	2	21 M, 24 F	9.0 (4.0)
	Term	21	28.2 (6.3)	39.3 (1.2)	3326.8 (652.7)	1	1	7 M, 14 F	11.5 (3.7)
	PTB	24	28.0 (5.9)^a^	34.9 (3.2)^b^	2822.5 (637.8)^c^	1	1	14 M, 10 F	6.8 (2.9)^a^
GAPPS Bangladesh	Total	80	23.9 (6.2)	37.4 (2.6)	2541.6 (546.5)	0	0	45 M, 35 F	5.3 (4.1)
	Term	45	23.0 (5.6)	39.4 (1.0)	2895.2 (284.7)			25 M, 20 F	4.3 (3.8)
	PTB	35	25.1 (6.8)^a^	34.8 (1.7)^b^	2087.0 (459.2)^b^			20 M, 15 F	6.5 (4.1)^a^
GAPPS Zambia	Total	60	29.0 (6.3)	36.7 (3.8)	2620.3 (757.7)	16	21	30 M, 28 F	8.4 (3.9)
	Term	31	29.2 (6.0)	39.6 (1.2)	3135.5 (445.5)	3	9	14 M, 15 F	6.4 (2.9)
	PTB	29	28.8 (6.8)^a^	33.6 (3.0)^b^	2069.7 (624.0)^b^	13	12	16 M, 13 F	10.5 (3.7)^a^
All sites combined	Total	341	25.7 (5.9)	37.0 (3.2)	2599.4 (612.4)	20	25	166 M, 173 F	6.7 (4.2)
	Term	177	25.7 (5.8)	39.4 (1.2)	2936.3 (428.2)	4	11	84 M, 91 F	6.8 (4.3)
	PTB	164	25.7 (6.1)^a^	34.4 (2.5)^b^	2211.3 (561.6)^b^	16	14	82 M, 82 F	6.6 (4.1)^a^

*AMANHI*, The Alliance for Maternal and Newborn Health Improvement; *GAPPS*, The Global Alliance to Prevent Prematurity and Stillbirth; *PTB*, preterm birth; *SD*, standard deviation.

*Robinson. Inflammation among placentas from Multi-Omics for Mothers and Infants sites. Am J Obstet Gynecol 2025.*

a Indicates not significant (PTB vs term)

b Indicates *P*<.01 (PTB vs term)

c Indicates *P*<.05 (PTB vs term).

**Supplemental Table 2:** Subject data for the participants whose placentas were used in transcriptomic analyses

Collection site	Subset	n	Maternal age (y)	Gestational age (wk)	Birth weight (g)	Inductions	Cesarean deliveries	Baby sex	Maternal education (y)
			Mean (SD)	Mean (SD)	Mean (SD)	#	#		Mean (SD)
AMANHI Bangladesh	Total	14	22.6 (4.2)	33.9 (4.5)	2224 (540)	0	0	9F, 5M	6.9 (2.3)
	Term	4	24.5 (12.3)	39.3 (1.0)	2555 (396)			3F, 1M	6.8 (2.4)
	PTB	10	21.9 (3.9)^a^	31.7 (3.3)^b^	2043 (541)^a^			6F, 4M	7.0 (2.4)^a^
AMANHI Pakistan	Total	14	26.4 (5.6)	37.4 (2.3)	2569 (368)	0	0	9F, 5M	3.1 (4.6)
	Term	7	29.7 (4.0)	39.3 (1.3)	2736 (329)			6F, 1M	4.0 (5.3)
	PTB	7	23.0 (5.1)^c^	35.5 (1.0)^b^	2401 (346)^a^			3F, 4M	2.1 (3.9)^a^
AMANHI Tanzania	Total	8	28.4 (6.4)	35.9 (1.0)	3059 (588)	2	2	3F, 5M	6.3 (3.1)
	Term	0	—	—	—	—	—	—	—
	PTB	8	28.4 (6.4)	35.9 (1.0)	3059 (588)	2	2	3F, 5M	6.3 (3.1)
GAPPS Bangladesh	Total	12	25.5 (5.8)	38.4 (2.4)	2648 (576)	0	0	5F, 7M	8.5 (1.4)
	Term	8	23.9 (5.4)	39.9 (0.6)	2909 (341)			3F, 5M	8.8 (1.0)
	PTB	4	28.8 (5.7)^a^	35.3 (1.5)^b^	2125 (631)^c^			2F, 2M	8.0 (2.2)^a^
GAPPS Zambia	Total	8	31.9 (6.9)	36.4 (2.4)	2423 (434)	1	2	5F, 3M	9.0 (4.5)
	Term	2	37.5 (2.1)	40.0 (0.8)	2796 (431)	0	1	2F, 0M	12.0 (4.2)
	PTB	6	30.0 (14.7)^a^	35.2 (0.8)^b^	2232 (227)^c^	1	1	3F, 3M	8.0 (4.4)^a^
All sites combined	Total	56	26.3 (6.2)	36.4 (3.3)	2557 (541)	3	4	31F, 25M	6.5 (3.9)
	Term	21	27.2 (6.1)	39.6 (1.0)	2792 (358)	0	1	16F, 9M	7.1 (4.2)
	PTB	35	25.8 (6.2)^a^	34.4 (2.6)^b^	2398 (590)^b^	3	3	15 F, 16M	6.1 (3.7)^a^

*AMANHI*, The Alliance for Maternal and Newborn Health Improvement; *GAPPS*, The Global Alliance to Prevent Prematurity and Stillbirth; *PTB*, preterm birth; *SD*, standard deviation.

*Robinson. Inflammation among placentas from Multi-Omics for Mothers and Infants sites. Am J Obstet Gynecol 2025.*

a Indicates not significant (PTB vs term)

b Indicates *P*<.01 (PTB vs term)

c Indicates *P*<.05 (PTB vs term).

**Supplemental Table 3:** List of the Taqman qRT-PCR probes used for validation studies

Gene symbol	Gene name	Taqman probe identifier
ANGPT2	Angiopoietin 2	Hs00169867_m1
ASB2	Ankyrin repeat and SOCS box containing	Hs00387867_m1
CXCL1	C-X-C motif chemokine ligand 1	Hs00236937_m1
CA1	Carbonic anhydrase 1	Hs00266139_m1
NFASC	Neurofascin	Hs00391791_m1
SERTAD4	SERTA domain containing 4	Hs00219702_m1

*qRT-PCR*, quantitative reverse transcriptase–polymerase chain reaction.

*Robinson. Inflammation among placentas from Multi-Omics for Mothers and Infants sites. Am J Obstet Gynecol 2025.*

## Supplementary Data

Supplementary Data 2

Supplemental Table 4 The MOMI data vs Paquette et al,^27^ 2023 The ECHO Prenatal and Early Childhood Pathways to Health consortium conducted a transcriptomic analysis of 48 preterm and 540 term placentas. They discovered 1729 genes that were differentially expressed (DE) in all the PTB cases (aPTB) when compared with term controls (corrected *P* value <.05). A total of 53 of these genes were also DE in the MOMI study. The Paquette analysis highlighted those that were DE in spontaneous early PTB (ePTB) or late PTB (lPTB) of which a subset also overlapped with those identified in the MOMI study. Red, up-regulated; green, down-regulated. *FC*, fold change; *MOMI*, Multi-Omics for Mothers and Infants; *PTB*, preterm birth. *Robinson. Inflammation among placentas from Multi-Omics for Mothers and Infants sites. Am J Obstet Gynecol 2024.*

Supplemental Table 5 The MOMI data vs Kheterpal et al, unpublished data In a study conducted by the Translation Health Sciences Institute of India in parallel with the MOMI analysis, placentas from PTB (n=20) and term births (n=13) were profiled using global RNA sequencing. In total, 20 genes were identified as being DE (Bonferonni adjusted *P*<.05; absolute fold change ≥1.5). Three were also identified in the MOMI study (names bolded). The table displays DE genes that were common between the 2 studies, the *P* values, and average fold change differences between PTB and term placentas. DE genes that were common between the MOMI and the Kheterpal study with a less conservative cutoff (uncorrected *P*<.05; absolute fold change ≥1.5) are also included. The figure displays the correlation in fold change differences between PTB and term births for the 3 highly significant DE genes identified in the 2 studies. Red, up-regulated; green, down-regulated. *DE*, differentially expressed; *FC*, fold change; *MOMI*, Multi-Omics for Mothers and Infants; *PTB*, preterm birth. *Robinson. Inflammation among placentas from Multi-Omics for Mothers and Infants sites. Am J Obstet Gynecol 2024.*
